# New York Letter

**Published:** 1881-06

**Authors:** 


### Domestic Correspondence.

---

### ARTICLE VIII.

### NEW YORK LETTER.

EDITORS CHICAGO JOURNAL AND EXAMINER :—I wrote in my last letter that the subject of mesmerism or trance had been assuming a good deal of prominence in various circles here. Of course the daily press takes up the matter, and makes as much sensation as possible out of a subject which contains plenty of material for exciting astonishment. There has been a good deal of exaggeration of the importance of this newly studied condition, but there have also been some uncalled-for attempts at ignoring it altogether. There can be no doubt of the reality of trance phenomena. Exactly how much they develop in ordinary life it is impossible yet to say. As far as the value of it to medical or surgical therapeutics is concerned, we may be quite sure it will amount to little in the hands of regular practitioners. We do know, however, that it is a powerful agent for effecting good and bad results with charlatans. I heard, only a little while ago, of a certain "doctor" who has an office on Sixth avenue, and who cures "by the laying on of hands." His method is to make the patient look fixedly at him while he makes certain passes over the head and face. He is reputed to have made many marvellous cures. During two hours in the morning he treats the poor, charging nothing. His office is then crowded with the credulous rich. Some time ago every one would have considered him not only an impostor, but one whose cures were all as imaginary as his pretensions were absurd. Now it can easily be understood that he probably does a good deal of his work by throwing the patients into a trance or trancoidal condition. The class of persons who visit him are just the kind that would be most susceptible. The methods employed by the "doctor" are also just those calculated to hypnotize the patients. This particular charlatan, therefore, only does what has been done here, experimentally, by regular practitioners.

Regarding the scientific value of trance, it is claimed that a study of it will throw much light upon some problems in physiology and psychology. One of the most interesting facts in connection with the former subject is the entire abolition of vertigo in the hypnotized subjects. Let any one fix his eye at a point on the ceiling; then turn around three or four times; then try to walk off in any direction. He will be sure either to stumble or fall. But hypnotize even an untrained subject, fix his attention on the ceiling in the same way, whirl him around, dehypnotize him at once, and he will walk off as steadily as any one could. Such an experiment certainly upsets some of the theories regarding the production of vertigo—particularly the one quite currently accepted, that it is produced in conditions like those above, by a movement of the endolymph in the semicircular canals, such movement exciting the auditory hairs that project into the fluid.

As regards psychology or physio-psychology, the promises of what a study of trance can contribute are still somewhat vague. There has recently been written a work, by Mr. Cyples, of London, containing some very elaborate studies of the methods by which the mind receives impressions and acquires experience. The work containing these studies has been indorsed as the best contribution to physio-psychology that has been made for many years. It is evident, however, that some of the conclusions of the author would have been modified, and some of his work improved, by a study of the curious phenomena of hypnotism.

I fear I am dwelling too long upon a subject that may not be thought worthy of so much attention. But I must add something in regard to the medico-legal relations of trance; for as far as one can tell now, this aspect of it is going to be the most important one.

At a meeting of the Medico-Legal Society, last April, Dr. W. H. Hammond introduced the subject of the possibility of persons, while in a trance state, doing criminal acts. He hypnotized a young man, and then, by various suggestions, made him commit an imaginary theft, a murder, and a forgery.

The experiments were well conceived, but too much was claimed for them by their author. His assertions were received with much incredulity, and even the reality of the condition of trance was denied. Dr. Hammond did not, he says, have a fair opportunity to defend his views. At any rate, the discussion was postponed till the next meeting. At this subsequent meeting, which occurred early in the present month, Dr. G. M. Beard occupied most of the time in presenting his view of the matter, and especially his theory of trance. The paper presented was a very good one, in its place, but the author dwelt upon his own views of trance, to the exclusion, largely, of its medico-legal relations.

Dr. Beard was, I believe, the first in this country to study thoroughly the subject, and he has probably spent more time and thought upon the matter than any one else. His digressions, therefore, might easily have been excused. They were not, however, by all of his audience. An amusing incident was caused by Dr. E. C. Spitzka, a gentleman who terrorizes with his logic and erudition the various medical societies to which he belongs. He called the speaker to order, making the point that he (the speaker) was going over facts that were old, out of place, and not on the announced order of the evening. The president ruled that the point of order was not well taken, whereupon Dr. Spitzka took his hat and marched out of the room. Now that Conkling and Platt have resigned because they could not have their own way, it seems that our great men are getting petulant. Dr. Beard, in course of time, came to the medico-legal part of his subject, and stated that he did not think that persons in trance could go through any complex acts, criminal or otherwise. They were like machines, and would respond to a suggestion, but would act only upon that. They could do simple acts, like stabbing, shooting, and injuring themselves or others. They might perform a simple act of theft. Acts of adultery might be unconsciously performed, or a woman might be impressed with the idea that she had been violated, just as occasionally occurs in cases of the administration of chloroform. A few other medico-legal points were referred to briefly, but the subject was not well brought out, and the subsequent discussion was very brief and of little importance.

Still, the matter has already got into the courts, not only in Paris, but in this country. The Whittaker case is asserted by Dr. Beard to be one of trance. This gentleman testified recently that the persons who mutilated Whittaker frightened him into a state of trance-coma, in which condition he was found. Other medical experts, such as Dr. L. C. Gray, have, to a certain extent, confirmed this opinion.

Any one would think, perhaps, from the local press of this city, that we were wading in garbage and cowering beneath the malign presence of half a dozen different pestilences. Our streets are rather dirty; we have some typhus fever, and rather more small-pox than usual. It is truly a disgrace that our streets are not cleaner, but the idea that the dirt in them is going to produce any violent epidemic is very extravagant. The mortality rate has not, on the whole, been much higher than usual, and has been lower than that of some other American cities. This would be especially true if the deaths of persons who have just reached the city—such as emigrants, or of transients in hospitals and elsewhere—were excluded. The cases of typhus fever are growing less, at date of writing, and the prospect is that the disease will die out. It has not been an easy thing to quarantine it, or to try and limit its spread. Thus, a short time ago, it came near infecting a whole infant asylum. A child was brought to the institution with the symptoms of scarlatina maligna. There was not much rash, but it had sore-throat, high fever, and great depression. The case was diagnosticated as one of scarlet fever, and, of course, put in the fever ward. Subsequent investigation, however, showed that it came from a house where two cases of typhus fever had been found. A closer study of the symptoms made it probable that the disease really was typhus. Fortunately, no one was infected. The present epidemic, indeed, has not been a severe one, as regards mortality or activity of infection.

Small-pox does not seem inclined to leave the city. The Board of Health has been very active in vaccinating the poor, and the vaccinating corps has done a great deal to limit the spread of the disease. Their work, this winter, has brought out a great deal of evidence of the protective power of vaccination, including some actually crucial cases. I mention this because we have in this city a small number of fanatics who have organized themselves into an anti-vaccination society. I cannot find that many converts have been made. When small-pox leaves the city, doubtless the membership will begin to increase. I notice that the *North American Review*, with its usual skill in hitting off topics of present interest, has in its June issue a short article by Dr. Austin Flint, Sr., on "Vaccination." The article is a brief and unpretentious one, containing nothing especially new on the subject.

The Faculty of the Bellevue Hospital Medical College have, it is said, been made terribly sore by the numerous adverse criticisms in the medical journals upon its course in going back to a two-term system. But there does not seem to be anything that can be done about it. The school will probably in time recover what prestige it had. It certainly has, after all, only shown a little moral weakness. It is just as good a college as its neighbor, the University—which is not saying so very much, to be sure. Some apologetic remarks for the latter college were recently made by Dr. St. John Roosa. He said that an endowment was necessary for a three-term graded course, and stated that the Harvard Medical School was enabled to adopt its present system because it was thus endowed. To this an answer came promptly back from Harvard, that such was not the case at all; that the college was not endowed at all, and that the change had been made at a considerable pecuniary sacrifice.

There is a rumor that a new weekly medical journal is to be started in this city. It is at first to rival, and finally supplant, the *Medical Record*, which now leads the race. The *Record*, it is said, does not contain enough original articles of a high scientific standard. Doubtless the independent attitude of the *Record* towards the various medical colleges here has something to do with the movement. The colleges have their rings, and are mostly close corporations; and what is a ring or a corporation without an organ? It is further rumored that $25,000 has been subscribed for the object in question. It is not stated, however, that the money has been paid in. And when the actual amount of work and money necessary for the conduct of a large weekly comes to be realized, the project will perhaps move more slowly.

Dr. L. A. Sayre has recently been indicted for malpractice. The plaintiff, a woman, alleges that Dr. Sayre gave her so much strychnine that it ruined her health. The case has not progressed very far yet, but it looks very much like one of blackmail.

Another interesting case recently in court was that of poisoning by a lemon meringue pie. Several persons who ate the pies made by a certain baker on a certain Saturday in October, 1879, were taken ill, with symptoms of gastro-intestinal irritation. One of the persons died, and the post-mortem showed that he had taken a corrosive poison. This was thought at first to be some salt of copper, produced by making the pie in copper kettles. Analysis showed, however, that there was no copper in the pie, but that there was a dye which stained wool yellow. This proved to be not the ordinary aniline yellow, which is probably innocuous, but dinitro-naphthol, a cheap yellow dye. This is the first case, probably, in which it has been found to have acted as a poison. The baker denied that he had put any dye in the pie, and it was not shown that he did it, or could have had any reason for doing it. However, the jury gave the plaintiff $1,000 damages. And persons who are fond of lemon meringue pies, if they go by the verdict, must look out for dinitro-naphthol in their dessert.

Our medical registration laws are now being tested, and, fortunately, the evidence in the two cases, or, at least, in one of them, is very strong. Two or three years ago, a man was called to account by the censors of the County Medical Society for practicing without any license. He then admitted that he had no degree, but protested against being interrupted in the pursuit of his living and the support of his family. Recently he has been caught trying to register his name as a regular practitioner, and claiming to have gotten his degree from the now defunct medical college at Castleton, Vt. The case is a clear one, and judgment was pronounced against him by the justice, but it has yet to be tested in the higher courts.

NEW YORK CITY, May 17, 1881.

